# Simulation OSEs (Observed Structured Educational Stations) to Develop Inter-Professional Education (IPE) and Collaborative Working (CW) in Healthcare at Cygnet Churchill Hospital London, UK

**DOI:** 10.1192/bjo.2025.10276

**Published:** 2025-06-20

**Authors:** Angela Misra, Omer Malik, Yugisha Gurung

**Affiliations:** Cygnet Healthcare, London, United Kingdom

## Abstract

**Aims:** Calhoun et al. assessed the effectiveness of in-situ simulation education and showed that adding in-situ simulation to current educational practices may improve patient mortality and morbidity.

Collaborative Working is when individuals with various professional backgrounds work together and combine their expertise with other individuals to provide the best care possible. The essence of collaborative working is “working as a team with respect and value for each team member’s unique role and contribution”.

The practice of interdisciplinary learning is already in place when Immediate Life Support simulation training is delivered. The concept of broadening this practice out to other areas of clinical practice is therefore not far-fetched and is evidence based.

**Aims:** Embed lessons learnt from serious untoward incidents within an simulated inter-professional educational environment; OSE implementation will improve staff ability to respond to clinical scenarios.

**Methods:** A total of 27 participants attended these sessions conducted across 2 days. The staff rotated through a four-station circuit (10 minutes each with 5 minutes verbal feedback): completing a NEWS2 chart (National Early Warning Score); recording glucose levels on a blood glucose monitoring chart; documenting neurological observations following Rapid Tranquillization administration and effective communication.

At each station, participants were asked to complete a Likert questionnaire to self-report their status in 4 key areas: Knowledge, Confidence, Management, Resource Awareness, and preference between Face-to-Face (F2F) or E-learning teaching. Following the station, participants received constructive feedback on their performance and repeated the questionnaire.

**Results:** Qualitative and quantitative data analysis was completed to assess the questionnaire responses, with all stations showing a significant increase in average scores (AS) across all Key Areas, ranging between 16.4% (NEWS2) and 54% (Neurological Observation).

Confidence in handling clinical scenarios showed the smallest AS increase across all stations ranging from 12.3% (NEWS2) to 46.1% (Neurological observation).

Awareness of trusted resources available had consistent minimal score change, with lowest score change of 6.1% observed in NEWS2. Post-session learning preferences strongly favoured F2F teaching, with all 27 participants preferring it for Communication and Blood Glucose monitoring stations. Neurological observation station showed the biggest improvements in knowledge (58.3%) and confidence in management (65.2%).

**Conclusion:** These findings highlight the importance of interactive teaching to improve clinical competency and knowledge retention. The data also suggests the need for improved resource awareness and accessibility and as a result resource folders have been disseminated. Cygnet Healthcare is also implementing OSEs across other hospital sites.

